# Chemical Stability of New Acyclovir Analogues with Peptidomimetics

**DOI:** 10.3797/scipharm.1012-20

**Published:** 2011-03-05

**Authors:** Georgi Hristov, Ivanka Stankova

**Affiliations:** Department of Chemistry, South-West University ‘Neofit Rilski’, Ivan Michailov str. 66, 2700 Blagoevgrad, Bulgaria

**Keywords:** Amino acids, Thiazole, Antiviral drugs, HPLC, Prodrugs

## Abstract

In the search for new and effective prodrugs against the herpes simplex virus, a series of acyclovir analogues with a thiazole ring containing amino acids (glycine, alanine, valine, leucine) has been investigated. The chemical stability of some of the compounds containing different residues was studied at pH 1 and pH 7.4 at a temperature of 37°C. An HPLC method was developed for quantification of the unchanged ester concentration.

Some of the esters (Gly-thiazole, Ala-thiazole-acyclovir, Leu-thiazole-acyclovir) were rather unstable, especially under acidic conditions, and underwent rapid hydrolysis into the chemical precursor acyclovir. At pH 7.4, the stability of Valthiazole-acyclovir was remarkable. At this pH, Val-thiazole-acyclovir showed stability higher than that of valacyclovir (the first effective prodrug of acyclovir).

## Introduction

The discovery of the guanine analogue of acyclovir (9-[(2-hydroxyethoxy)methyl]guanine), ACV, set the stage for a new generation of antiviral agents [1, 2]. Along with its high specificity, ACV demonstrates low aqueous solubility and low bioavailability by oral administration (14%). To overcome this problem, several acyclovir amino acid esters have been synthesised. Valaciclovir, the valine ester of ACV, was found to metabolise easily by oral administration and possess four-fold higher bioavailability than acyclovir [3–7].

Recently, we synthesised acyclovir esters with peptidomimetics and studied their antiviral activity against HSV-1 and HSV-2 [8–10].

Contrary to acyclovir esters with natural amino acids, the results showed that modification of acyclovir with thiazole, oxazole and thiazolyl-thiazole containing amino acids (Gly, Val, Ala, Leu) decreased the antiviral effect of acyclovir. The only exception with prominent activity against HSV-2 turned out to be the Ala-thiazolyl ester of acyclovir [8, 9]. The object of this study was to assess the chemical stability of some of the synthesised acyclovir esters with peptidomimetics at pH 1.0 and pH 7.4 at 37°C. An HPLC method was used for quantification of the ester concentrations [11, 12].

## Results and Discussion

**Fig. 1.**
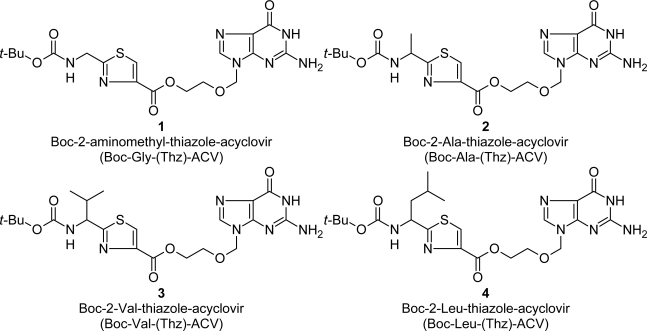
Acyclovir esters with peptidomimetics.

The chemical stability of acyclovir esters: Boc-2-aminomethyl-thiazole-acyclovir (**1**), Boc-2-Ala-thiazole-acyclovir (**2**), Boc-2-Val-thiazole-acyclovir (**3**) and Boc-2-Leu-thiazole-acyclovir (**4**) was studied under experimental conditions of biological relevance, i.e. at pH 1 and pH 7.4, at a temperature of 37°C. The compounds were synthesised as previously described [8, 9]. The structures of the compounds under investigation are presented in Fig. 1.

It was established that, under the described experimental conditions, some esters underwent decomposition by hydrolysis. The hydrolysis followed apparent first order kinetics, and the rate constants (K) were obtained as slopes from the semi-logarithmic plots of the unchanged ester concentration versus time. The chemical stability was assessed by means of the decomposition half-lives:

$$t_{1/2}=\text{ln}\frac{2}{K}$$

**Fig. 2.**
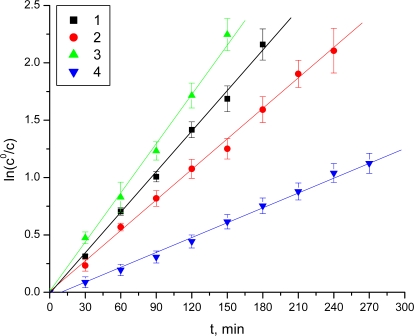
Decrease of the concentration of the examined prodrugs at pH 1.0 (HCl)

Chemical stability measurements revealed that the dipeptide prodrugs of acyclovir were relatively unstable at acidic pH (Tab. 1, Fig. 2). Under these conditions, all of the observed t_½_ were less than 2.6 h.

**Fig. 3.**
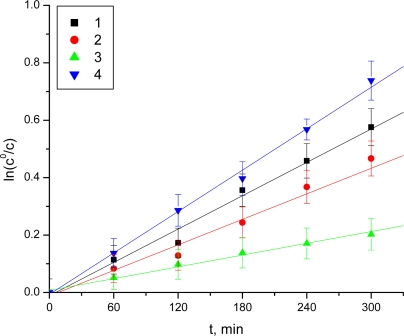
Decrease of the concentration of the examined prodrugs in buffer solution at pH 7.4 (phosphate buffer)

At pH 7.4, the Boc-2-Val-thiazole-acyclovir (**3**) was more stable (t_½_ = 17 h) than valacyclovir (t_½_ = 13 h) [3], the first effective prodrug of acyclovir, (Tab. 1, Fig. 3).

The Boc-2-Val-thiazole-acyclovir (**3**) and Boc-2-aminomethyl-thiazole-acyclovir (**1**) were less stable than the Boc-2-Ala-thiazole-acyclovir (**2**) and Boc-2-Leu-thiazole-acyclovir (**4**) at pH 1.0. The esters (**1**) and (**4**) manifested lower stability at pH 7.4. It was found that the rest of compounds were stable at pH 7.4.

## Experimental

### Chemicals

Acetonitrile for HPLC the buffer components HCI, Na_2_H_2_PO_4_ of the purest grade, were purchased from Merck (Germany). The Grace Vidac chromatographic column was used (USA).

### Chromatography

Chromatography was carried out isocratically, on a modular KNAUER HPLC system (Germany), consisting of a Smartline Pump 1000, a Smartline Manager 5000 solvent degasser, an injector with a 20 μl loop and a Smartline UV Detector 2600 diode array. The analyses were controlled and the data were acquired with EuroChrom software. The mobile phase consisted of acetonitrile/water in a ratio of 30:70 or 50:50 v/v depending on the polarity of the compound and a flow rate 1 ml/min was used. The detection was performed at relevant λ_max_ for the respective compound (range 252–262 nm).

### Kinetic study

A single chromatographic method was used to detect the studied acyclovir esters with thiazole rings containing amino acids (Gly, Ala, Val, Leu) in aqueous buffer solutions at pH 1.0 (0.1 M HCl) and pH 7.4 (phosphate buffer). Twenty microlitres of each sample were injected into a reverse phase HPLC C18 column. The mobile phase consisted of acetonitrile/water at a ratio of 30:70 or 50:50 v/v depending on the polarity of the compound. The analyses of the esters of acyclovir with amino acid (Gly, Ala, Val, Leu) containing thiazole rings were validated. The specificity of the method was investigated by observing potential interference between the esters of acyclovir and its parent drug, acyclovir. No interfering peaks were presented in the chromatograms. The linearity of the relationship between the peak area and concentration was determined by analysing six standard solutions in a concentration range of 0.1–1.0 mmol/l. For all analytes, the relationship between the peak area ratio of the drug to the internal standard and concentration was linear over the entire examined concentration range. The correlation coefficients of the calibration curves were greater than 0.997. For all of the examined compounds the coefficient of variation calculated for the six analysed samples did not exceed 5%.

Hydrolysis of acyclovir esters with thiazole rings containing amino acids (Gly, Ala, Val, Leu) was studied at pH 1.0 (HCl) and pH 7.4 (phosphate buffered saline). Stock solutions of the prodrugs were prepared and used immediately for stability studies. Aliquots (9.8 ml) of the buffer were placed in a screw-capped vial and allowed equilibrate at 37°C. A prodrug stock solution (0.2 ml) was added to the buffer. The vial was placed in a constant shaker bath set at 37°C and 60 rpm. Each sample was directly analysed by HPLC.

## Conclusion

The chemical stability of acyclovir esters with peptidomimetics was studied under experimental conditions simulating some relevant biological media (pH 1.0 and pH 7.4, 37°C). The examined compounds were not stable in acidic media. This result was predictable, because the compounds are esters and esters easily undergo decomposition by hydrolysis in acidic media. The order of decreasing stability in neutral media and at a temperature of 37°C was 3>>2>4>1. Under the same conditions, the Boc-2-Val-thiazole-acyclovir (**3**) was more stable than valacyclovir (t_½_ = 13 h), the first effective prodrug of acyclovir [4]. Boc-2-Val-thiazole-acyclovir (**3**) exhibit the highest chemical stability at pH 7.4 compared to the other examined compounds, but it did not exhibit a satisfying effect against HSV-1 or HSV-2 [5]. The compound Boc-2-Ala-thiazole-acyclovir (**2**) showed an appreciable effect against HSV-2 and exhibited satisfying chemical stability. The correlation with both biological activity and chemical stability suggests that the Boc-2-Ala-thiazole-acyclovir (**2**) could be attractive for antiviral chemotherapy.

## Figures and Tables

**Tab. 1.** The *t*_½_ values of the examined compounds at pH=1 and pH=7.4

**№**	**Compound**	**pH=1, *t***_½_**, h**	**pH=7.4, *t***_½_**, h**
1	Boc-Gly(Thz)-ACV	1.1±0.1	5.9±0.5
2	Boc-Ala(Thz)-ACV	1.3±0.1	7.8±0.5
3	Boc-Val(Thz)-ACV	0.7±0.1	17.8±0.9
4	Boc-Leu(Thz)-ACV	2.6±0.2	5.0±0.4
